# Polysaccharides from Hemp Seed Protect against Cyclophosphamide-Induced Intestinal Oxidative Damage via Nrf2-Keap1 Signaling Pathway in Mice

**DOI:** 10.1155/2020/1813798

**Published:** 2020-08-25

**Authors:** Ran Xue, Ming Du, Tian-Yi Zhou, Wan-Zheng Ai, Zhong-Shan Zhang, Xing-Wei Xiang, Yu-Fang Zhou, Zheng-Shun Wen

**Affiliations:** ^1^Zhejiang Provincial Engineering Technology Research Center of Marine Biomedical Products, School of Food Science and Pharmaceutics, Zhejiang Ocean University, Zhoushan 316022, China; ^2^Key Laboratory of Vector Biology and Pathogen Control of Zhejiang Province, Huzhou University, Huzhou Cent Hosp, Huzhou 313000, China; ^3^Department of Food Science and Engineering, Zhejiang University of Technology, Hangzhou 310014, China; ^4^Zhejiang Marine Development Research Institute, Zhoushan, Zhejiang 316021, China

## Abstract

Hemp seed has been used as a traditional oriental medicine and health food in China for centuries. Polysaccharides from hemp seed (HSP) exhibit important properties of intestinal protection, but there are limited data on the specific underlying mechanism. The primary objective of this study was to investigate the protective effect of HSP on intestinal oxidative damage induced by cyclophosphamide (Cy) in mice. The results showed that pretreatment with HSP significantly increased the average daily gain, thymus index, spleen index, superoxide dismutase (SOD), catalase (CAT), and glutathione peroxidase (GSH-Px) activity in serum and ileal homogenate and significantly reduced malondialdehyde (MDA) content in ileal homogenate. In addition, the expression levels of SOD, GSH-Px, Nrf2, heme oxidase-1 (HO-1), and quinoneoxidoreductase-1 (NQO1) mRNA in ileal homogenate were significantly increased. Western blot results showed that HSP significantly upregulated the expression of Nrf2 protein and downregulated the expression of Keap1 protein in the ileum. Collectively, our findings indicated that HSP had protective effects on intestinal oxidative damage induced by Cy in mice, and its mechanism might be related to the activation of Nrf2-Keap1 signaling pathway.

## 1. Introduction

Oxidative stress is defined as the imbalance between oxygen free radicals and antioxidants [1]. Oxygen free radicals usually contain reactive oxygen species (ROS) [2]. The intestine is an essential organ and the main place for digestion and absorption. However, the intestinal tissue as important barrier is easy to be attacked by stressful conditions such as oxidative stress, resulting in an increase in permeation of toxins. When there is excessive accumulation of oxygen free radical, they may damage the intestinal mucosal barrier, increase intestinal mucosal permeability, and disorder intestinal flora in the intestine, thereby affecting the body's steady state system [3, 4]. The previous study has shown that oxidative stress was a major factor causing several tissue injuries in intestinal ischemia and reperfusion (I/R) [5]. Dalsing et al. found that intestinal ischemia was improved largely by pretreating oxygen radical scavengers (dimethylsulfoxide and superoxide dismutase) [6]. In addition, oxidative stress is believed to be an important factor in the pathogenesis of intestinal inflammation [7–9]. There are evidences that the administration of antioxidants is essential for the prevention and treatment of colorectal cancer [10, 11].

Cyclophosphamide (Cy) is used as a chemotherapy drug for cancer treatment. It is inactive in vitro, but it can effectively kill cells in the proliferative cycle under the action of liver microsomal enzymes in vivo [12]. Cy kills tumor cells as well as some rapidly proliferating normal tissue cells (gastrointestinal mucosal cells, hepatocytes, etc.) [13]. Furthermore, Cy was found to have a variety of side effects such as weaking body resistance, decreasing immune function, and inducing oxidative damage, especially damage of the gastrointestinal mucosal barrier [14–17]. Therefore, Cy was selected as an inducer to establish intestinal oxidative damage model in mice in the present study.

Hemp seed is the fructus of the hemp (Cannabis sativa L.) plant and is mainly distributed in China. Hemp seed has been commonly used as a Chinese traditional medicine and food for the persons from Bama Yao Autonomous County, Guangxi province, China. Some studies investigated the potential protective effects of hemp seed on many diseases. Lu et al. found that hemp seed proteins protected PC12 cells from H_2_O_2_-induced oxidative damage and apoptosis [18]. Girgih et al. found that hemp seed protein hydrolysate (HPH) had higher metal chelation activity and potential to treat diseases such as oxidative stress [19]. Previous studies reported that cold-pressed hemp seed oil had strong free radical scavenging activity and oxidative stability *in vitro* [20]. Moreover, our previous study found the polysaccharide of hemp seed (HSP, a sulfated polysaccharide) displayed good antioxidant activity *in vitro* [21]. Investigations on hemp seed suggest that it has potential as an alternative treatment for oxidative stress-related diseases. Some studies indicated that sulfated polysaccharides derivatives showed better free radical scavenging activities [22, 23]. However, further investigation is needed to identify the exact mechanism of action and pathways that was modulated by HSP *in vivo*. Therefore, in the present study, the protective effect of HSP on intestinal oxidative damage and its related mechanisms were elucidated using mouse cyclophosphamide-induced intestinal oxidative damage model and provided a scientific evidence for further development of HSP as a natural intestinal antioxidant protection in the food and pharmaceutical industries.

## 2. Materials and Methods

### 2.1. Experimental Animals

Healthy male ICR mice, weighing 18–22 g, 6-8 weeks old, procured from the Zhejiang Academy of Medical Sciences (Hangzhou, Zhejiang, China) were used in this study. All mice were fed with sterile water and a commercial pellet diet *ad libitum* for acclimatization for 3 days before starting the experiment. The mice were housed and maintained at constant room (temperature: 23 ± 1°C, air humidity: 55% ± 5%) under a regular light/dark schedule (12 h/12 h).

### 2.2. Chemicals and Reagents

Cyclophosphamide (Cy) was purchased from Aladdin Chemistry Co. Ltd. (Shanghai, China). Malondialdehyde (MDA), catalase (CAT), superoxide dismutase (SOD), and glutathione peroxidase (GSH-Px) assay kits were purchased from Nanjing Jiancheng Bioengineering Institute (Nanjing, China). The antibodies against Nrf2 and Keap1 were acquired from Abcam (Cambridge, UK). Monoclonal antibody against *β*-actin was from Santa Cruz Biotechnology, Inc. (Santa Cruz, CA, USA). All other reagents were of the highest grade or of analytical grade available commercially.

### 2.3. Preparation of Polysaccharides from Hemp Seeds (HSP)

Polysaccharides from hemp seeds (HSP) were extracted and purified by our laboratory. Briefly, defat hemp seed was extracted by distilled water at 60°C for 2 h. The supernatant was collected, concentrated, and added to ethanol. After being kept at 4°C overnight, the supernatant was removed by centrifuging for 10 min at 3000 rpm. The precipitate was collected and dissolved in distilled water to remove the protein by Sevag method [24]. In brief, the polysaccharide solution and the Sevag reagent (chloroform: n-butanol =4 : 1, *v*/*v*) were mixed (3 : 1, *v*/*v*) and shaken vigorously for 30 min at room temperature and centrifuged at 3000 × g for 10 min. The water layers were collected, and we repeated the above operation until the protein layers were gone. The polysaccharide was prepared after dialyzed and lyophilized. The content of polysaccharide was dissolved in distilled water.

### 2.4. Compositional Analysis

The FT-IR determination used a Bruker Tensor II (Bruker, Germany) to analyze organic functional groups in the frequency range of 4000-500 cm^−1^. The molecular weight of the HSP sample was measured by HPGPC. The monosaccharide composition of HSP was determined according to the precolumn derivatization with 1-phenyl-3-methyl-5-pyrazolone (PMP) high-performance liquid chromatography method [24]. The sulfate contents of HSP sample was determined according to a published literature [21].

### 2.5. Treatment of Animals

The mice (*n* = 10/group) were randomly divided into four groups according to body weight. Mice were administered with HSP by gavage for 21 consecutive days and Cy by intraperitoneal injection (I.P.) for 3 days (from day 18 to 21) (Table 1). The clinical symptoms and weight of mice were monitored daily and recorded. At the end of this study, the mice were sacrificed by eye enucleation for collecting blood. The serum was obtained after collected blood clotting, and then centrifuged with 2000 rpm for 10 min, and stored at -80°C until assay. The thymus, spleen, and intestine were removed immediately on an ice-cold plate and stored at -80°C until assay.

### 2.6. Measurement of Organ Index

At the end of this study, all mice were sacrificed at the 21th day, the thymus and spleen were taken and weighed. Then, the organ index was measured according to the following formula [25]:

(1) $$\begin{array}{c} \text{Organ Index}=\text{organ weight}\left(\text{mg}\right)/\text{body weight}\left(\text{g}\right)\times 10. \end{array}$$

### 2.7. Ultrastructural Observation of Jejunum in Mice

Mouse intestinal tissues were sampled and fixed by immersion in precooled 2.5% glutaraldehyde at 4°C overnight for scanning electron microscopy. Briefly, the sample fixed in 1% osmium tetroxide for 1 h. Then, they were washed with 0.1 M sodium cacodylate and dehydrated with a series of ethanol 50%, 70%, 80%, 95%, and 100%. They were immersed in hexamethyldisilazane (HMDS) for 15 min three times. The jejunum samples were air dried, mounted, and coated with gold. Finally, the intestinal tissues were measured with the SU8010 Scanning Electron Microscopy (SEM).

### 2.8. Detection of Antioxidant Enzyme Activity in Serum

The activities of the antioxidant enzymes including superoxide dismutase (SOD), glutathione peroxidase (GSH-Px), and catalase (CAT) in serum were determined according to the manufacturer's instructions of the kit (Nanjing Jiancheng bioengineering institute, Nanjing, Jiangsu, China). The levels of SOD was measured at 550 nm using the hydroxylamine method with 1510 spectrophotometer (Thermo Fisher Scientific Oy, Vantaa, Finland), and the levels of GSH-Px and CAT were assayed by colorimetric method at 405, 412 nm, respectively.

### 2.9. Detection of Antioxidants Status in Ileum Tissues

A sample of 100 mg of ileum tissue was homogenized in 1 mL of 0.9% normal saline under an icy environment. The 10% ileum homogenates were centrifuged at 3,000 rpm for 10 min at 4°C, and the supernatants were separated by decantation for the measurement of antioxidant levels. Antioxidant levels including SOD, GSH-Px, CAT, and MDA were detected in 10% ileum homogenates. Antioxidant enzymes SOD, GSH-Px, and CAT activities and MDA content were determined according to the manufacturer's instructions of the commercial kits (Nanjing Jiancheng bioengineering institute, Nanjing, Jiangsu, China).

### 2.10. Quantitative Real-Time Polymerase Chain Reaction

Total RNA of ileum was extracted with 1 mL ice-cold TRIzol reagent (Ambion, Carlsbad, USA). The quality and concentration of total RNA were quantified by spectrophotometry at 260 and 280 nm. Then, the synthesis of the first strand of cDNA was achieved using a PrimeScript 1st Strand cDNA Synthesis Kit (Takara, Dalian, Japan). The primer sequences were obtained from Shanghai Shenggong Biotechnology Co., Ltd. (Shanghai, China) and listed in Table 2. The relative mRNA expression of the target gene was measured as described previously [21]. The target genes mRNA expression were analyzed by the 2^-*ΔΔ*Ct^ method and normalized to the mean values of internal reference gene (*β*-actin). Each sample was performed in triplicate.

### 2.11. Western Blot Analysis

RIPA Lysis Buffer was used to prepare lysates of ileum. The concentration of proteins from the ileum was analyzed by using BCA Protein Assay Kit (Beyotime, Shanghai, China). About 30 *μ*g protein samples were separated using SDS-PAGE (10%) and transferred onto 0.45 *μ*m polyvinylidene difluoride (PVDF) membranes (Merck Millipore, Massachusetts, USA). After blocking (5% nonfat milk (BD, New Jersey, USA)), followed by the membranes incubated with primary antibodies (Abcam, Cambridge, UK)) at 4°C overnight, the membranes were washed three times with TBS for 5 min. Subsequently, the membranes were incubated with the HRP-conjugated secondary antibody for 1 h at room temperature and washed three times with TBST for 5 min. Chemiluminescence imaging of protein were performed with ECL agents (Beyotime, Shanghai, China). The band density was analyzed with ImageJ software. The ratio of the proteins examined was normalized against *β*-actin. Each sample had three triplicate.

### 2.12. Statistical Analysis

Date were expressed as mean ± standard error of the mean (SEM) and analyzed by the one-way ANOVA procedure of SPSS 23.0 software. Duncan's test was adopted to determine significant differences among multiple groups, and significant differences were designated as *p* less than 0.05.

## 3. Results

### 3.1. Structural Features

As shown in Table 3, the monosaccharide composition of HSP was composed of Man, Rha, GlcUA, GalN, Gal, Xyl, and Ara in a molar ratio of 6.85 : 4.94 : 3.85 : 22.19 : 1.76 : 35.26 : 25.16, respectively. The result suggested that HSP was heteropolysaccharide. The sulfate content and molecular weight of HSP were 1.48% and 42.1 kDa.

**Figure 1:**
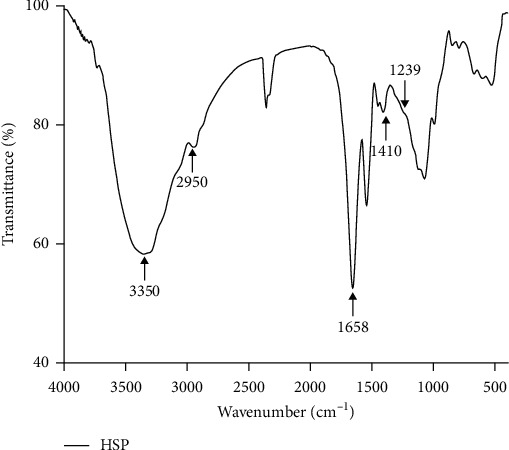
FT-IR spectra of HSP.

The FT-IR spectra of HSP were shown in Figure 1. The characteristic absorption bands around 3350 cm^−1^ and 2950 cm^−1^ attributed to –OH and –CH stretching vibration. The characteristic peaks at around 1658 cm^−1^ was the C=O stretching vibration. The absorption frequencies appear at 1410 cm^−1^, which is a characteristic of the uronic acids. Bands at 1239 cm^−1^ was assigned to the asymmetric O=S=O stretching vibration of sulfate esters.

### 3.2. Effect of HSP on Body Weight and Organ Index

**Figure 2:**
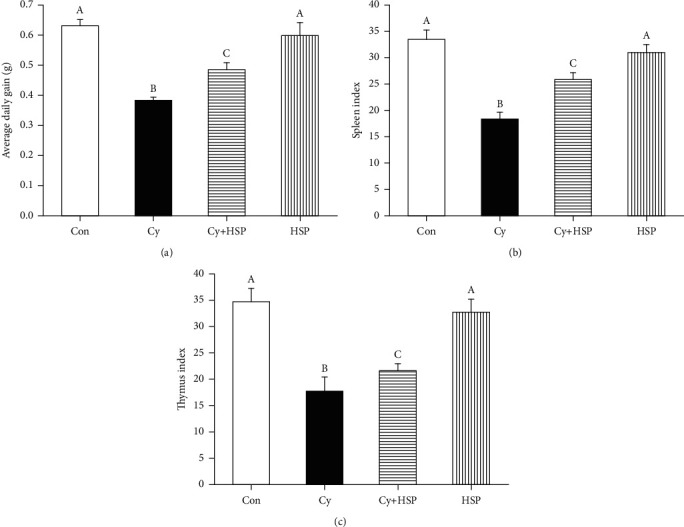
Effect of hemp seed polysaccharide on average daily gain (a), spleen index (b), and thymus index (c) in immunosuppressed mice. Each bar represents mean ± SEM (*n* = 10). Bars with different letters (A, B, and C) are statistically different (*p* < 0.05).

The results of average daily gain, thymus index, and spleen index were shown in Figure 2. Cy significantly reduced the average daily gain, thymus index, and spleen index of mice compared with the control group (*p* < 0.05). Compared with the Cy-treated group, HSP-pretreated group significantly increased the average daily gain (Figure 2(a)), spleen index (Figure 2(b)), and thymus index (Figure 2(c)) (*p* < 0.05). At the same time, there was no significant difference in the organ indexes (thymus and spleen index) of HSP-treated group compared with the Con group.

### 3.3. Effect of HSP on Ultrastructure of Jejunum

**Figure 3:**
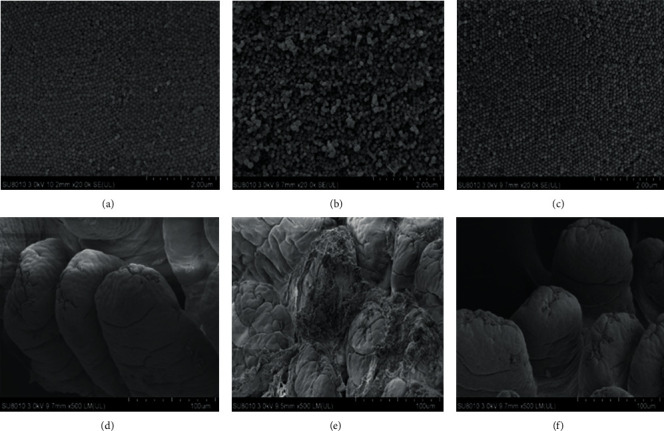
Effect of HSP on ultrastructure of jejunum. (Con (a), Cy (b), and Cy+HSP (c), SEM 20x; Con (d), Cy (e), and Cy+HSP (f), SEM 500x).

The results of the scanning electron microscope were shown in Figure 3. The villi were arranged neatly in order with the same shape and size and smooth surface (Figure 3(d)), moreover, the Con group had smooth and complete microvilli, arranged neatly and tightly (Figure 3(a)). In the Cy group, the villi was severely damaged with uneven surface (Figure 3(b)), and the microvilli irregularly distributed, partially defective, and collapse and atrophy were found (Figure 3(e)). Microscopy of the Cy+HSP group showed that the lesions were alleviated, the villi surface was not damaged, and the whole was relatively flat (Figure 3(c)). Additionally, the surface of microvilli was recovered, and the collapse and atrophy was decreased (Figure 3(f)).

### 3.4. Effect of HSP on Antioxidant Enzyme Activity in Serum

**Figure 4:**
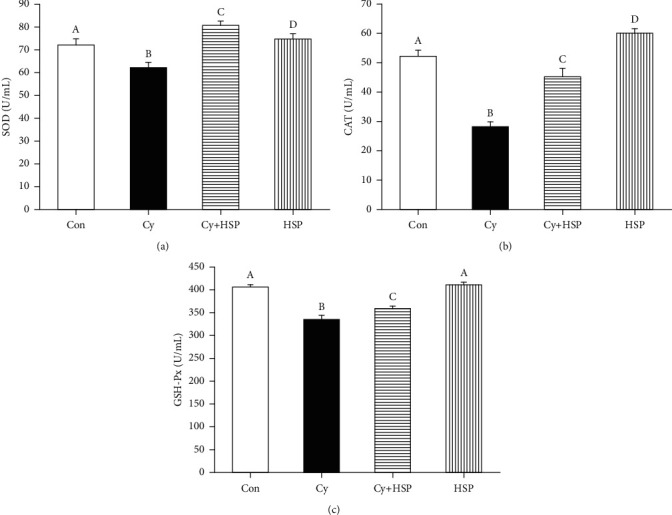
Effect of hemp seed polysaccharide on serum antioxidant levels SOD (a), CAT (b), and GSH-Px (c) in mice. Each bar represents mean ± SEM (*n* = 10). Bars with different letters (A, B, C, and D) are statistically different (*p* < 0.05).

The effect of HSP on the antioxidant enzyme levels of mice was evaluated and shown in Figure 4. Compared with the Con group, Cy significantly decreased the activity of SOD (Figure 4(a)), CAT (Figure 4(b)), and GSH-Px (Figure 4(c)) in the serum of mice (*p* < 0.05). Compared with the Cy-treated group, HSP significantly increased the activity of SOD, CAT, and GSH-Px in the serum of mice (*p* < 0.05). Meanwhile, HSP significantly increased the serum CAT activity (*p* < 0.05), but there was no significant difference in serum SOD and GSH-Px activities compared with the Con group (*p* > 0.05). The results showed that HSP enhanced the antioxidant capacity of mice and reduced the oxidative stress induced by Cy.

### 3.5. Effect of HSP on Antioxidant Stress Levels in Ileum Tissues

**Figure 5:**
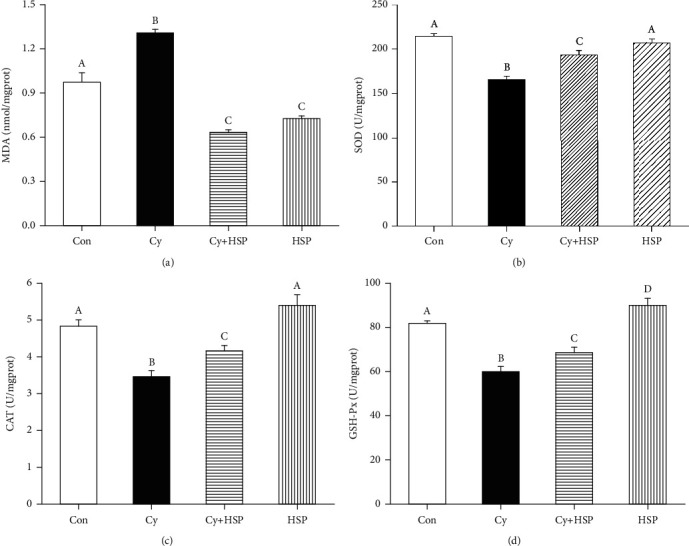
Effects of hemp seed polysaccharide on the antioxidant levels MDA (a), SOD (b), CAT (c), and GSH-Px (d) in the ileum of mice. Each bar represents mean ± SEM (*n* = 10). Bars with different letters (A, B, C, and D) are statistically different (*p* < 0.05).

The effect of HSP on the antioxidation level in ileum tissues of mice were evaluated and shown in Figure 5. Compared with the Con group, Cy significantly decreased SOD (Figure 5(b)), CAT (Figure 5(c)), and GSH-Px (Figure 5(d)) activities and significantly increased MDA levels (Figure 5(a)) in the ileum of mice (*p* < 0.05). Compared with the Cy-treated group, HSP-pretreated group significantly increased the activity of SOD, CA,T and GSH-Px and significantly decreased the MDA content in the ileum of mice (*p* < 0.05). Meanwhile, the HSP-treated group significantly increased the activity of GSH-Px and decreased the MDA content in the ileum (*p* < 0.05) but had no significant effect on the activity of SOD and CAT in the ileum compared with the Con group (*p* > 0.05).

### 3.6. Effect of HSP on the Expression Level of Related Antioxidant Genes in Ileum

**Figure 6:**
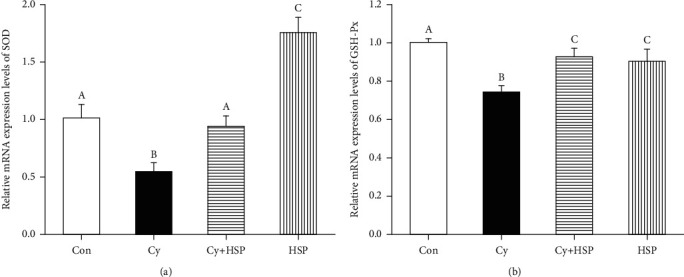
Effect of hemp seed polysaccharide on the expression levels of antioxidant genes SOD (a) and GSH-Px (b) in the ileum of mice. Each bar represents mean ± SEM (*n* = 10). Bars with different letters (A, B, and C) are statistically different (*p* < 0.05).

The effect of HSP on the expression levels of antioxidant genes in the ileum of mice were evaluated and indicated in Figure 6. Compared with the Con group, Cy significantly inhibited the expression levels of SOD (Figure 6(a)) and GSH-Px (Figure 6(b)) mRNA in the ileum of mice (*p* < 0.05). Compared with the Cy-treated group, the HSP-pretreated group remarkably increased the expression levels of SOD and GSH-Px mRNA in the ileum of mice (*p* < 0.05). Meanwhile, the HSP-treated group significantly increased the expression of SOD mRNA (*p* < 0.05) and had no significant effects on the expression of GSH-Px mRNA in the ileum of mice (*p* > 0.05).

### 3.7. Effect of HSP on the Expression Level of Nrf2 and Phase 2 Detoxifying Genes in Ileum

**Figure 7:**
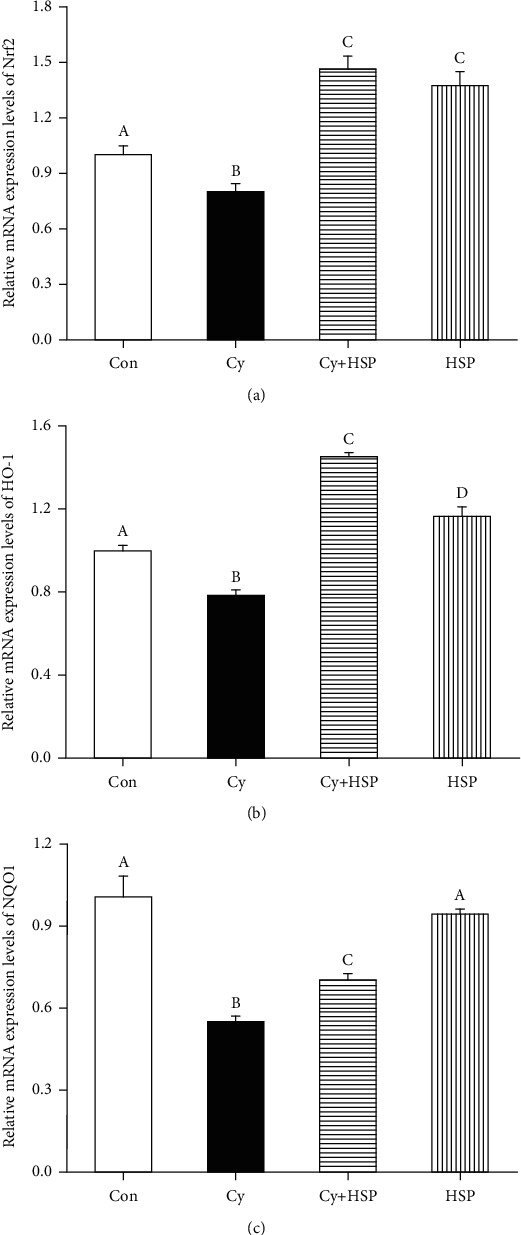
Effect of hemp seed polysaccharide on the expression levels of the relative genes Nrf2 (a), HO-1 (b), and NQO1 (c) in ileum of mice. Each bar represents mean ± SEM (*n* = 10). Bars with different letters (A, B, C, and D) are statistically different (*p* < 0.05).

The effect of HSP on the expression level of Nrf2 and phase 2 detoxifying genes in the ileum of mice was investigated, and results were shown in Figure 7. Compared with the Con group, Cy significantly decreased the expression levels of Nrf2 (Figure 7(a)), HO-1 (Figure 7(b)), and NQO1 (Figure 7(c)) mRNA in the ileum of mice (*p* < 0.05). Compared with the Cy-treated group, the HSP-pretreated group remarkably increased the expression levels of Nrf2, HO-1, and NQO1 mRNA in the ileum of mice (*p* < 0.05). Moreover, the HSP-treated group significantly increased the expression of Nrf2 and HO-1 mRNA (*p* < 0.05) and had no significant effects on the expression levels of NQO1 mRNA in the ileum of mice compared with the Con group (*p* > 0.05).

### 3.8. Effect of HSP on Nrf2-Keap1 Signaling Pathway

**Figure 8:**
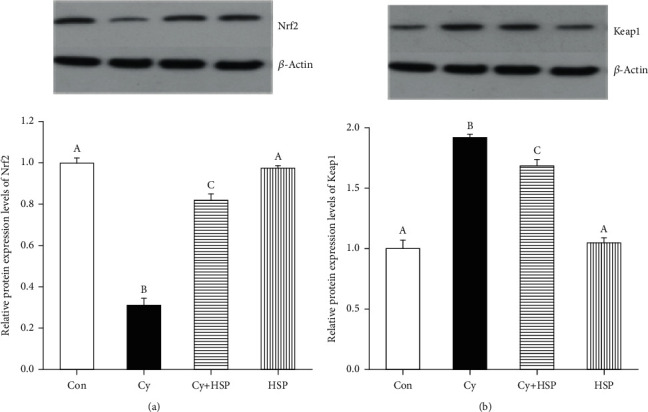
Effect of hemp seed polysaccharide on the Nrf2/Keap1 signaling pathway in the mouse ileum. (a) The whole tissue lysates were prepared and used for the Nrf2 and *β*-actin western blot analysis. (b) The whole tissue lysates were prepared and used for the Keap1 and *β*-actin western blot analysis. Each bar represents mean ± SEM (*n* = 10). Bars with different letters (A, B, C, and D) are statistically different (*p* < 0.05).

To evaluate the effect of HSP on the Nrf2-Keap1 signaling pathway, the expression levels of Nrf2 and Keap1 protein were detected and results were shown in Figure 8. Compared with the Con group, Cy significantly decreased the expression of Nrf2 protein (Figure 8(a)) and increased the expression of Keap1 protein (Figure 8(b)) in the ileum of mice (*p* < 0.05). Compared with the Cy-treated group, the HSP-pretreated group remarkably increased the expression levels of Nrf2 (Figure 8(a)) and inhibited the expression of Keap1 protein (Figure 8(b)) in the ileum of mice (*p* < 0.05). At the same time, the HSP-treated group had no significant effect on the expression of Nrf2 and Keap1 protein in the ileum of mice compared with the Con group (*p* > 0.05).

## 4. Discussion

Cyclophosphamide (Cy) is commonly used as a chemotherapeutic drug in clinics [26]. Cy exerts antineoplastic effects and also causes side effects such as gastrointestinal mucosal damage [17]. In recent years, polysaccharides isolated from botanical sources have received great attention due to their low toxicity and strong antioxidant ability [27–29]. *Anoectochilus roxburghii* polysaccharides (ARP) possessed a hepatoprotective effect against CCl4-induced acute liver damage via reducing lipid oxidation [30]. A pectic polysaccharide extracted from rhizome of *L*. *chuanxiong* (LCP-II-I) enhanced the intestinal antioxidant defense capacity of aged mice via enhancing the expression level of antioxidant enzymes [31]. In this study, we established an acute intestinal mucosal oxidative injury model in mice by intraperitoneal injection of Cy and studied the protective effect of HSP on intestinal mucosal oxidative damage by preadministration of HSP. In the present study, the results showed that the body weight of the mice was significantly decreased after four days of intraperitoneal injection of Cy.

The spleen and thymus are important immune organs of the body. The previous studies have shown that Cy could induce generation of oxidative stress with reduction in the index of the spleen and thymus in mice [32–34]. Previous studies reported that polysaccharides increased immune organ indexes (spleen index and thymus index), suggesting that polysaccharides have a protective effect on immune organs of body [22, 34, 35]. Our results showed that Cy significantly reduced the thymus index and spleen index of mice. However, administration of HSP significantly increased the thymus index and spleen index of mice, suggesting that HSP could prevent the atrophy of the thymus and spleen. The results indicated that HSP had a protective effect on the immune organs in Cy-induced oxidative damage mice.

Superoxide dismutase (SOD), glutathione peroxidase (GSH-Px), and catalase (CAT) are important components of antioxidant defense systems [36]. Malondialdehyde (MDA) is the final product of lipid peroxidation and is also one of the markers of oxidative stress [37, 38]. In addition, the intestine is the key organ involved in the body's nutrient absorption and metabolism [39]. Therefore, we measured the antioxidant levels in the serum and ileal tissue. In the present study, the SOD, CAT, and GSH-Px activities were dramatically decreased and the MDA level was increased after stimulation with Cy for 4 days, implying that superfluous ROS was induced on mice. HSP significantly increased the activity of SOD, CAT, and GSH-Px in the serum and ileum tissues and also remarkably decreased the MDA content in the ileum compared with the Cy treatment group. This results indicated that HSP had a preventive protective effect against Cy-induced intestinal oxidative damage by enhancing the antioxidant enzyme activity and reducing the lipid peroxidation level in the intestine. Similar results have been reported in previous studies that plant polysaccharides protected mice from oxidative damage by Cy. Yu et al. found that *Ganoderma atrum* polysaccharide (PSG-1) significantly increased the total antioxidant capacity and activities of SOD, CAT, and GSH-Px and decrease the MDA level in mice [40]. Cui et al. reported that Polygonum Cillinerve (Nakai) Ohwi crude polysaccharides (PCCP) had protective effects against oxidative damage in immunosuppressed ICR mice induced by Cy [41]. Therefore, this study demonstrated that HSP was able to protect mice against Cy-induced oxidative damage. Our previous study found the fraction HSP0.2 from HSP dramatically increased the activities of SOD, GSH-Px, and CAT and decreased the level of MDA in IPEC-1 cells model induced by hydrogen peroxide [21]. Some studies found that polysaccharides showing strong antioxidant activity might be related to the sulfate group [42–44].

SOD and GPx are the major enzymes responsible for the inactivation of superoxide and hydrogen peroxide, respectively [45]. In order to clearly establish that antioxidant enzymes were involved in the antioxidant activity of HSP, we determined the mRNA expression levels of antioxidant enzymes in ileal tissues. The results showed that HSP significantly increased the levels of SOD and GSH-Px mRNA in ileal tissues, which was consistent with the results of measurements of antioxidant enzymes SOD and GSH-Px levels. A previous study found Ganoderma lucidum polysaccharide derivatives increased SOD and GSH-Px levels in mice; moreover, sulfated polysaccharides showed better antioxidant activity than other polysaccharide derivatives [22]. Our findings suggested that HSP regulated the activity of antioxidant enzymes via enhancing the mRNA expression level of intestinal antioxidant enzymes, thereby alleviating the intestinal damage caused by oxidative stress. The results of the study indicated that HSP as a sulfated polysaccharides could enhance expression of a variety of antioxidant enzymes to defend oxidative damage as the previous studies [21, 46, 47].

NF-E2-related factor 2 (Nrf2) is a key transcription factor that regulates antioxidant responses [48]. Under conditions of oxidative stress, Nrf2 binds to the antioxidant response element (ARE), thereby activating antioxidant enzymes and phase II detoxification enzyme expression [49, 50]. Heme oxygenase-1 (HO-1) and NADPH quinone oxidoreductase 1 (NQO1) are important downstream target genes of the Nrf2 signaling pathway and play key roles in response to oxidative-stress-mediated injury [51, 52]. Real-time quantitative PCR results showed that HSP significantly increased the expression of Nrf2 mRNA in the ileum and subsequently upregulated the expression levels of related detoxification enzymes HO-1 and NQO1 as downstream antioxidant genes of the Nrf2 signaling pathway. It is concluded that the antioxidant effects of HSP might be related to the activation of the Nrf2 signaling pathway. Tripathi et al. reported that melatonin treatment significantly increased in the Nrf2 level as well as associated phase-II enzymes NQO-1 and HO-1 in oxidative damage induced by Cy in mice [53]. Additionally, we detected the expression of Nrf2 and Keap1 proteins with western blot. As our results indicated, treatment with Cy led to a reduction in the Nrf2 protein expression which was in accordance with the results of some previous reports. Yang et al. found that *Lycium barbarum* polysaccharide (LBP) has a protective effect against Cy-induced ovarian injury by reducing oxidative stress and activating the Nrf2/ARE signaling pathway [54]. Le et al. reported that squid ink polysaccharide (SIP) could effectively relieve testicular damage induced by Cy through the Nrf2/ARE signal pathway [55]. HSP pretreatment significantly upregulated the expression of Nrf2 protein in the ileum and inhibited the expression of Keap1 protein. Our previous study found that the fraction HSP0.2 from HSP had a protective effect against hydrogen peroxide-induced oxidative stress in IPEC-1 cells through the regulation of the Keap1/Nrf2 signaling pathway [21]. This results further confirmed that HSP had a protective effect on Cy-induced intestinal oxidative damage in mice, and its mechanism may be related to the activation of the Nrf2-Keap1 signaling pathway.

## 5. Conclusions

The present study indicated that HSP significantly increased the average daily gain, thymus index, and spleen index of mice induced by cyclophosphamide (Cy). Moreover, HSP remarkably improved SOD, CAT, and GSH-Px levels and significantly decreased the MDA level in the serum and intestinal tissue. In addition, HSP could significantly increase the mRNA expression levels of SOD, GSH-Px, Nrf2, HO-1, and NQO1 and upregulated the expression of Nrf2 protein and downregulated the expression of Keap1 protein in the ileum. Taken together, our findings indicated that HSP could have protective effects on intestinal oxidative damage induced by Cy in mice, and its mechanism may be related to the enhancement of endogenous antioxidant activity, gene expression of antioxidant enzymes, and the activation of the Nrf2-Keap1 signaling pathway. These results suggested that HSP had a potential in the treatment of intestinal oxidative damage and other related diseases.

## Figures and Tables

**Table 1:** Mice experimental grouping.

Groups	HSP (1-21th day)	Cy (18, 19, 20, and 21th day)
Con	Distilled water	0.9% normal saline
Cy	Distilled water	50 mg cy/kg BW/day
Cy+HSP	200 mg HSP/kg BW/day	50 mg cy/kg BW/day
HSP	200 mg HSP/kg BW/day	0.9% normal saline

Con: control; Cy: cyclophosphamide; Cy+HSP: cyclophosphamide+hemp seed polysaccharide; HSP: hemp seed polysaccharide.

**Table 2:** Oligonucleotide primers used in quantitative real-time polymerase chain reaction.

Gene	Gene accession number	Primer sequence 5′-3′	Product size (bp)
SOD	NM_011434.1	F: ATGGCGATGAAAGCGGTGTG	465
		R: TTACTGCGCAATCCCAATCACTC	
GPx	NM_ 008160.6	F: GCAATCAGTTCGGACACCAG	126
		R: CACCATTCACTTCGCACTTCTC	
Nrf2	NM_ 010902	F: TTCCTCTGCTGCCATTAGTCAGTC	215
		R: GCTCTTCCATTTCCGAGTCACTG	
HO-1	NM_010442.2	F: GATAGAGCGCAACAAGCAGAA	111
		R: CAGTGAGGCCCATACCAGAAG	
NQO1	NM_008706.5	F: GGACATGAACGTCATTCTCT	261
		R: TTCTTCTTCTGCTCCTCTTG	
*β*-Actin	NM_007393	F: AGTGTGACGTTGACATCCGT	298
		R: GCAGCTCAGTAACAGTCCGC	

F: forward; R: reverse.

**Table 3:** The monosaccharide composition of HSP.

Sample		Monosaccharide composition (Mol %)					
	Man	Rha	GlcUA	GalN	Gal	Xyl	Ara
HSP	6.85	4.94	3.85	22.19	1.76	35.26	25.16

^∗^Man: mannose; Rha: rhamnose; GlcUA: glucuronic acid; GalN: galacturonic acid; Gal: galactose; Xyl: xylose; Ara: arabinose.

## Data Availability

The data used to support the findings of this study are included within the article.

## References

[1] Bhimaraj A., Tang W. H. W. (2012). Role of oxidative stress in disease progression in stage B, a pre-cursor of heart failure. *Heart Failure Clinics*.

[2] Valko M., Rhodes C. J., Moncol J., Izakovic M., Mazur M. (2006). Free radicals, metals and antioxidants in oxidative stress-induced cancer. *Chemico-Biological Interactions*.

[3] Sun Z., Liu W., Gao W. (2010). Identification and characterization of the dominant lactic acid bacteria from kurut: the naturally fermented yak milk in Qinghai, China. *Journal of General & Applied Microbiology*.

[4] Zhang Y., du R., Wang L., Zhang H. (2010). The antioxidative effects of probiotic Lactobacillus casei Zhang on the hyperlipidemic rats. *European Food Research and Technology*.

[5] Turan I., Ozacmak H. S., Ozacmak V. H., Barut F., Araslı M. (2017). Agmatine attenuates intestinal ischemia and reperfusion injury by reducing oxidative stress and inflammatory reaction in rats. *Life Science*.

[6] Dalsing M. C., Grosfeld J. L., Shiffler M. A. (1983). Superoxide dismutase: a cellular protective enzyme in bowel ischemia. *Journal of Surgical Research*.

[7] Karp S. M., Koch T. R. (2006). Oxidative stress and antioxidants in inflammatory bowel disease. *Disease.-a-month*.

[8] Mulder T. P., Verspaget H. W., Janssens A. R., de Bruin P. A., Pena A. S., Lamers C. B. (1991). Decrease in two intestinal copper/zinc containing proteins with antioxidant function in inflammatory bowel disease. *Gut*.

[9] Markowitz M. M., Rozen P., Pero R. W., Tobi M., Miller D. G. (1998). Hydrogen peroxide induced adenosine diphosphate ribosyl transferase (ADPRT) response in patients with inflammatory bowel disease. *Gut*.

[10] Gackowski D., Banaszkiewicz Z., Rozalski R., Jawien A., Olinski R. (2002). Persistent oxidative stress in colorectal carcinoma patients. *International Journal of Cancer*.

[11] Chang D., Wang F., Zhao Y.-S., Pan H.-Z. (2008). Evaluation of oxidative stress in colorectal cancer patients. *Biomedical and Environmental Sciences*.

[12] Cohen J. L., Jao J. Y. (1970). Enzymatic basis of cyclophosphamide activation by hepatic microsomes of the rat. *Journal of Pharmacology and Experimental Therapeutics*.

[13] Farrell C. L., Bready J. V., Rex K. L. (1998). Keratinocyte growth factor protects mice from chemotherapy and radiation-induced gastrointestinal injury and mortality. *Cancer Research*.

[14] Milas L., McBride W. H., Ito H., Hunter N. (1984). WR-2721 protects antitumor immune resistance against damage by whole body irradiation (WBI) and cyclophosphamide (CY). *International Journal of Radiation Oncologybiologyphysics*.

[15] Haque M. R., Ansari S. H., Rashikh A. (2013). Coffea arabica seed extract stimulate the cellular immune function and cyclophosphamide-induced immunosuppression in mice. *Iranian Journal of Pharmaceutical Research*.

[16] Sadir S., Deveci S., Korkmaz A., Oter S. (2007). Alpha-tocopherol, beta-carotene and melatonin administration protects cyclophosphamide-induced oxidative damage to bladder tissue in rats. *Cell Biochemistry and Function*.

[17] Shi H., Chang Y., Gao Y. (2017). Dietary fucoidan of Acaudina molpadioides alters gut microbiota and mitigates intestinal mucosal injury induced by cyclophosphamide. *Food and Function*.

[18] Lu R. R., Qian P., Sun Z. (2010). Hempseed protein derived antioxidative peptides: purification, identification and protection from hydrogen peroxide-induced apoptosis in PC12 cells. *Food Chemistry*.

[19] Girgih A. T., Udenigwe C. C., Li H., Adebiyi A. P., Aluko R. E. (2011). Kinetics of enzyme inhibition and antihypertensive effects of hemp seed (Cannabis sativa L.) protein hydrolysates. *Journal of the American Oil Chemists Society*.

[20] Prescha A., Grajzer M., Dedyk M., Grajeta H. (2014). The antioxidant activity and oxidative stability of cold-pressed oils. *Journal of the American Oil Chemists Society*.

[21] Wen Z.-S., Xue R., du M. (2019). Hemp seed polysaccharides protect intestinal epithelial cells from hydrogen peroxide-induced oxidative stress. *International Journal of Biological Macromolecules*.

[22] Wang J., Wang Y., Liu X., Yuan Y., Yue T. (2013). Free radical scavenging and immunomodulatory activities of Ganoderma lucidum polysaccharides derivatives. *Carbohydrate Polymers*.

[23] Wen Z., Liu L., OuYang X., Qu Y., Chen Y., Ding G. (2014). Protective effect of polysaccharides from Sargassum horneri against oxidative stress in RAW264.7 cells. *International Journal of Biological Macromolecules*.

[24] Zhu K., Zhang Y., Nie S. (2017). Physicochemical properties and in vitro antioxidant activities of polysaccharide from Artocarpus heterophyllus Lam. Pulp. *Carbohydrate Polymers*.

[25] Sun J. H., Liu Y. M., Cao T., Ouyang W. Q. (2013). Effect of kinetin on ovary and uterus in D-galactose-induced female mouse model of aging. *Acta Physiologica Sinica*.

[26] Sowjanya B. L., Devi K. R., Madhavi D. (2009). Modulatory effects of garlic extract against the cyclophosphamide induced genotoxicity in human lymphocytes in vitro. *Journal of Environmental Biology*.

[27] Zhao W., Jiang X. J., Deng W. W., Lai Y. H., Wu M., Zhang Z. Z. (2012). Antioxidant activities of Ganoderma lucidum polysaccharides and their role on DNA damage in mice induced by cobalt-60 gamma-irradiation. *Food and Chemical Toxicology*.

[28] Yang X., Yang S., Guo Y., Jiao Y., Zhao Y. H. (2013). Compositional characterisation of soluble apple polysaccharides, and their antioxidant and hepatoprotective effects on acute CCl4-caused liver damage in mice. *Food Chemistry*.

[29] Tan P. X., Tao Y. E., Liu X. X., He J. H. (2010). Research advances in antioxidant composition of botanical extracts and their action mechanisms. *Food Science*.

[30] Yang Z., Zhang X., Yang L. (2017). Protective effect of Anoectochilus roxburghii polysaccharide against CCl_4_-induced oxidative liver damage in mice. *International Journal of Biological Macromolecules*.

[31] Huang C., Cao X., Chen X. (2017). A pectic polysaccharide from Ligusticum chuanxiong promotes intestine antioxidant defense in aged mice. *Carbohydrate Polymers*.

[32] Yu J., Chen Y., Zhai L. (2015). Antioxidative effect of ginseng stem-leaf saponins on oxidative stress induced by cyclophosphamide in chickens. *Poultry Science*.

[33] Wei X. J., Hu T. J., Chen J. R., Wei Y. Y. (2011). Inhibitory effect of carboxymethylpachymaran on cyclophosphamide-induced oxidative stress in mice. *International Journal of Biological Macromolecules*.

[34] Chen J., Hu T., Zheng R. (2007). Antioxidant activities of Sophora subprosrate polysaccharide in immunosuppressed mice. *International Immunopharmacology*.

[35] Chen J., Zhu X. Q., Yang L. (2016). Effect of Glycyrrhiza uralensis Fisch polysaccharide on growth performance and immunologic function in mice in Ural City, Xinjiang. *Asian Pacific Journal of Tropical Medicine*.

[36] Ighodaro O. M., Akinloye O. A. (2018). First line defence antioxidants-superoxide dismutase (SOD), catalase (CAT) and glutathione peroxidase (GPX): their fundamental role in the entire antioxidant defence grid. *Alexandria Journal of Medicine*.

[37] Jiang J., Zhuang J. Y., Fan Y. Y., Shen B. (2009). Mapping of QTLs for leaf malondialdehyde content associated with stress tolerance in rice. *Rice Science*.

[38] Gaweł S., Wardas M., Niedworok E., Wardas P. (2004). Malondialdehyde (MDA) as a lipid peroxidation marker. *Wiadomosci Lekarskie*.

[39] Zeuzem S. (2000). Gut-liver axis. *International Journal of Colorectal Disease*.

[40] Yu Q., Nie S. P., Wang J. Q. (2014). Chemoprotective effects of Ganoderma atrum polysaccharide in cyclophosphamide-induced mice. *International Journal of Biological Macromolecules*.

[41] Cui J. J., Yuan J. F., Zhang Z. Q. (2010). Anti-oxidation activity of the crude polysaccharides isolated from Polygonum cillinerve (Nakai) Ohwi in immunosuppressed mice. *Journal of Ethnopharmacology*.

[42] Shao P., Chen X., Sun P. (2014). Chemical characterization, antioxidant and antitumor activity of sulfated polysaccharide from Sargassum horneri. *Carbohydrate Polymers*.

[43] Ananthi S., Raghavendran H. R., Sunil A. G., Gayathri V., Ramakrishnan G., Vasanthi H. R. (2010). In vitro antioxidant and in vivo anti-inflammatory potential of crude polysaccharide from Turbinaria ornata (Marine Brown Alga). *Food and Chemical Toxicology*.

[44] Yang Y., Liu D., Wu J., Chen Y., Wang S. (2011). In vitro antioxidant activities of sulfated polysaccharide fractions extracted from Corallina officinalis. *International Journal of Biological Macromolecules*.

[45] Spanier G., Xu H., Xia N. (2009). Resveratrol reduces endothelial oxidative stress by modulating the gene expression of superoxide dismutase 1 (SOD1), glutathione peroxidase 1 (GPx1) and NADPH oxidase subunit (Nox4). *Journal of Physiology and Pharmacology*.

[46] Ahmed A. A., Fedail J. S., Musa H. H., Musa T. H., Sifaldin A. Z. (2016). Gum arabic supplementation improved antioxidant status and alters expression of oxidative stress gene in ovary of mice fed high fat diet. *Middle East Fertility Society Journal*.

[47] Herrera M., Retanaugalde R., Gallegos J. L., Narvaez V. (2010). Pyrimethamine induces oxidative stress in plasmodium yoelii 17XL-infected mice: a novel immunomodulatory mechanism of action for an old antimalarial drug?. *Experimental Parasitology*.

[48] Li W., Kong A. N. (2010). Molecular mechanisms of Nrf2-mediated antioxidant response. *Molecular Carcinogenesis*.

[49] Lee J. S., Surh Y. J. (2005). Nrf2 as a novel molecular target for chemoprevention. *Cancer Letters*.

[50] Cho H. Y., Reddy S. P., Debiase A., Yamamoto M., Kleeberger S. R. (2005). Gene expression profiling of NRF2-mediated protection against oxidative injury. *Free Radical Biology and Medicine*.

[51] Zhang Z., Qu J., Zheng C. (2018). Nrf2 antioxidant pathway suppresses Numb-mediated epithelial-mesenchymal transition during pulmonary fibrosis. *Cell Death and Disease*.

[52] Ning S., Sekar T. V., Scicinski J. (2015). Nrf2 activity as a potential biomarker for the pan-epigenetic anticancer agent, RRx-001. *Oncotarget*.

[53] Tripathi D. N., Jena G. B. (2010). Effect of melatonin on the expression of Nrf2 and NF-kappaB during cyclophosphamide-induced urinary bladder injury in rat. *Journal of Pineal Research*.

[54] Yang D. M., Zhang J. Q., Fei Y. F. (2017). Lycium barbarum polysaccharide attenuates chemotherapy-induced ovarian injury by reducing oxidative stress. *Journal of Obstetrics and Gynaecology Research*.

[55] Le X., Luo P., Gu Y., Tao Y. E., Liu H. (2015). Squid ink polysaccharide reduces cyclophosphamide-induced testicular damage via Nrf2/ARE activation pathway in mice. *Iranian Journal of Basic Medical Sciences*.

